# Estimating community-wide indirect effects of influenza vaccination: triangulation using mathematical models and bias analysis

**DOI:** 10.1093/aje/kwae365

**Published:** 2024-09-17

**Authors:** Nimalan Arinaminpathy, Carrie Reed, Matthew Biggerstaff, Anna T Nguyen, Tejas S Athni, Benjamin F Arnold, Alan Hubbard, Art Reingold, Jade Benjamin-Chung

**Affiliations:** MRC Centre for Global Infectious Disease Analysis, School of Public Health, Imperial College, London SW7 2AZ, United Kingdom; Influenza Division, National Center for Immunization and Respiratory Diseases, Centers for Disease Control and Prevention, Atlanta, GA 30341, United States; Influenza Division, National Center for Immunization and Respiratory Diseases, Centers for Disease Control and Prevention, Atlanta, GA 30341, United States; Department of Epidemiology and Population Health, School of Medicine, Stanford University, Stanford, CA 94035, United States; Department of Epidemiology and Population Health, School of Medicine, Stanford University, Stanford, CA 94035, United States; Francis I. Proctor Foundation and Department of Ophthalmology, University of California, San Francisco, San Francisco, CA 94158, United States; Division of Epidemiology & Biostatistics, School of Public Health, UC Berkeley, Berkeley, CA 94720, United States; Division of Epidemiology & Biostatistics, School of Public Health, UC Berkeley, Berkeley, CA 94720, United States; Department of Epidemiology and Population Health, School of Medicine, Stanford University, Stanford, CA 94035, United States; Chan Zuckerberg Biohub, San Francisco, CA 94158, United States

**Keywords:** influenza, vaccines, indirect effects, mathematical modeling, probabilistic bias analysis, triangulation

## Abstract

Understanding whether influenza vaccine promotion strategies produce community-wide indirect effects is important for establishing vaccine coverage targets and optimizing vaccine delivery. Empirical epidemiologic studies and mathematical models have been used to estimate indirect effects of vaccines but rarely for the same estimand in the same data set. Using these approaches together could be a powerful tool for triangulation in infectious disease epidemiology because each approach is subject to distinct sources of bias. We triangulated evidence about indirect effects from a school-located influenza vaccination program using 2 approaches: a difference-in-difference (DID) analysis and an age-structured, deterministic, compartmental model. The estimated indirect effect was substantially lower in the mathematical model than in the DID analysis (2.1% [95% Bayesian credible intervals, 0.4%-4.4%] vs 22.3% [7.6%-37.1%]). To explore reasons for differing estimates, we used sensitivity analyses and probabilistic bias analyses. When we constrained model parameters such that projections matched the DID analysis, results only aligned with the DID analysis with substantially lower preexisting immunity among school-age children and older adults. Conversely, DID estimates corrected for potential bias only aligned with mathematical model estimates under differential outcome misclassification. We discuss how triangulation using empirical and mathematical modeling approaches could strengthen future studies.

## Introduction

Vaccination is critical for the prevention of seasonal influenza morbidity and mortality, particularly among vulnerable groups such as the elderly.^1^ In the United States and elsewhere, recommendations for seasonal influenza vaccination include all age groups^2^ older than 6 months of age. However, vaccine coverage for schoolchildren—the age group that contributes most to influenza transmission^3^—has remained below target levels.^4^

Promoting vaccination among schoolchildren through school-located influenza vaccination (SLIV) programs has been found to produce community-wide indirect effects (ie, reductions in influenza among individuals who did not participate in such programs). In prior studies, indirect effect sizes varied widely.^5-10^ Accurate estimates of indirect effects are important for establishing whether SLIV programs should be scaled up.

Vaccine indirect effects are a function of vaccine coverage levels, population immunity, community transmission, contact patterns, and the population age distribution. While these parameters are typically fixed in empirical epidemiologic studies (eg, cohort studies, trials), mathematical models can be used to identify influential drivers of indirect effects under hypothetical scenarios. Both methods have been used to estimate indirect effects of influenza vaccines,^3^^,^^5-13^ but rarely have they been used for the same estimand in the same data set.

We explored drivers of indirect effects using triangulation,^14^^,^^15^ a method of drawing stronger causal inferences using multiple methods with different sources of bias (Table 1).

**Table 1:** Overview of analytic approaches, sources, and directions of bias used in triangulation.

**Approach**	**Data analyzed**	**Description**	**Assumptions**	**Hypothesized sources and directions of bias**
Cohort study with difference-in-difference analysis	Rates of laboratory-confirmed influenza hospitalization in the intervention and comparison sites in the preintervention period (2011-2013) and intervention period (2017-2018)	Quasi-experimental approach that compares changes in outcomes over time between an intervention group and a comparison group to account for preintervention differences in outcome rates between groups	Preintervention outcome trends are parallel between intervention and comparison groups No common shocks influencing both intervention and comparison groups that are independent of the intervention Independence of the intervention and outcome at baseline No spillover effects between intervention and comparison groups or change in composition of the groups during the follow-up period	*Outcome misclassification* Incomplete testing for influenza among hospitalized patients, assuming same testing rate between sites (bias toward the null) Incomplete testing for influenza among hospitalized patients, assuming differing testing rates between sites (bias in unknown direction) *Unmeasured time-dependent confounding* Higher insurance rate, vaccination, or better overall health in intervention site during vs preintervention; assume insurance reduces hospitalization risk (bias away from the null) Higher insurance rate, vaccination, or better overall health in control site during vs preintervention; assume insurance reduces hospitalization risk (bias toward the null)
Compartmental mathematical model	Rates of laboratory-confirmed influenza hospitalization in the intervention and comparison sites in the intervention period (2017-2018)	A type of mathematical model that divides a population into compartments (eg, susceptible, infected, and recovered individuals) and tracks transitions between compartments over time to model the spread of diseases within a population	Age-specific mixing governed by self-reported contacts and drawn from the literature: otherwise, homogeneous mixing of individuals in the study population Compartmentalization of the population into susceptible, infected (asymptomatic), infected (symptomatic), and recovered/immune individuals Constant transmission parameters over the course of the epidemic Effect of vaccination is to reduce susceptibility to infection Homogeneous infectivity of infected individuals, regardless of vaccination status	Underestimated contact rate between schoolchildren and elderly in the intervention site (bias toward the null) Overestimated preexisting immunity to circulating strain among schoolchildren and the elderly (bias toward the null) Overestimated duration of infection and underestimated infectiousness (bias toward the null) Underestimated proportion symptomatic and infectiousness of asymptomatic cases (bias toward the null)

Mathematical models are constructed to reflect mechanistic processes of disease transmission within a population and are then calibrated to real-world data. To be tractable, mathematical models must make simplifications of reality (eg, assuming random mixing) that may introduce bias. As a result, different mathematical models may produce different results, even when applied to the same question.^16^^,^^17^ On the other hand, empirical epidemiologic studies (eg, cohort studies or trials) fit a statistical model to quantify relationships in data without specifying any mechanistic processes. While epidemiologic studies may better capture complex real-world transmission dynamics, they may be subject to unmeasured confounding, exposure or outcome misclassification, and selection bias.

We triangulated evidence about indirect effects from a city-wide school-located influenza vaccination program using a difference-in-difference analysis^10^ and an age-structured, deterministic, compartmental model. To inform inferences, we used sensitivity analyses and probabilistic bias analyses.

## Methods

### Study population and intervention

The Shoo the Flu program (https://www.shootheflu.org) was delivered in Oakland, California, offering delivery of free influenza vaccinations at elementary schools (kindergarten through grade 5). The intervention was initiated in 2014; here, we focused on the 2017-2018 season, which had the highest incidence of influenza and the largest estimate of intervention effectiveness in the cohort study. In 2014-2015 and 2015-2016, the live attenuated influenza vaccines that were primarily delivered through the program had low effectiveness; in 2016-2017, influenza transmission levels were lower.^18^ In the 2017-2018 season, the program delivered quadrivalent inactivated influenza vaccinations to 7536 of 34 741 eligible students in 95 elementary schools.

### Overview of triangulation approaches

**Figure 1:**
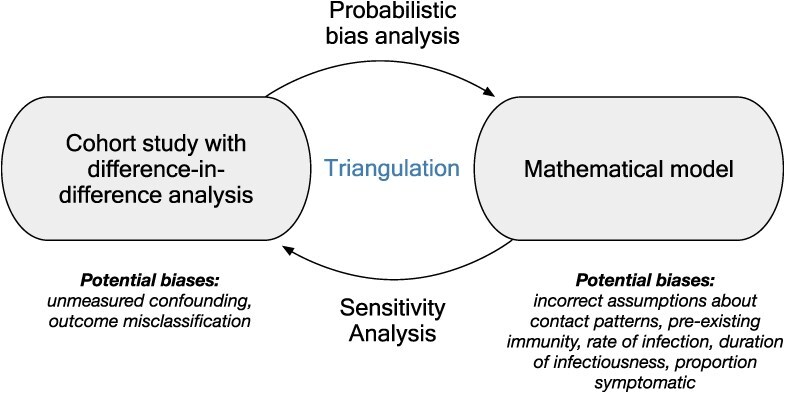
Triangulation approach. We triangulated evidence about indirect effects from a school-located influenza vaccination program using 2 approaches: a difference-in-difference (DID) analysis and an age-structured, deterministic, compartmental model. We used probabilistic bias analysis to attempt to reconcile difference-in-difference estimates with mathematical model results and used sensitivity analyses to reconcile mathematical model results with those of the difference-in-difference analysis.

We used 4 methods to triangulate evidence about indirect effects of school-located influenza vaccination among adults $\ge$65 years of age (Figure 1, Table 1). (1) Difference-in-difference analyses, which compare changes in outcomes over time between an intervention group and a comparison group; they can be used in quasi-experimental studies to account for preintervention differences in outcome rates between groups. (2) Compartmental models, a type of mathematical model to simulate disease transmission that divides a population into compartments (eg, susceptible, infected, and recovered individuals) and tracks transitions between compartments over time to model the spread of diseases. (3) To triangulate the findings of the difference-in-difference analysis with those of the compartmental model, probabilistic bias analyses, which assess the influence of biases upon effect estimates while incorporating statistical uncertainty with the goal of enhancing the overall validity of epidemiologic findings.^19^ (4) To triangulate the findings of the compartmental modeling with those of the difference-in-difference analysis, sensitivity analyses, which vary the key parameters or assumptions of a model to quantify their influence on model outcomes, providing insights into model predictions’ robustness and reliability.

### Difference-in-difference analysis

In a previous study, we empirically estimated the indirect effect of the intervention using a difference-in-difference analysis of data from a cohort study that enrolled 34 schools in the Oakland Unified School District and 34 schools in the West Contra Costa Unified School District (the comparison group).^10^ Intervention and comparison schools were pair-matched by preintervention school-level characteristics (mean enrollment, class size, parental education, academic performance index scores, California standardized test scores, school-level percentage of English language learners, and school-level percentage of students receiving free lunch at school) using a multivariate genetic matching algorithm.^20^ The study conducted a survey of parents and guardians of students to estimate influenza vaccination coverage. The study estimated indirect effects using data on laboratory-confirmed influenza hospitalizations among residents aged 65 years or older from the ZIP codes that overlapped with the boundaries of each school district, as reported by the Centers for Disease Control and Prevention (CDC)–sponsored California Emerging Infections Program (CEIP). We controlled for preintervention differences between districts using difference-in-difference models, which estimated the difference in incidence in the intervention district before the intervention and during the intervention minus the difference in incidence in the comparison district before and during the intervention. We fitted a log-linear Poisson model with a log population size offset and adjusted for age, sex, and race. The model included an interaction term between an indicator for the intervention group and an indicator for the time period before vs during the intervention (2011-2013 vs 2017-2018). Additional details on the intervention and cohort study design have been reported elsewhere.^10^

### Probabilistic bias analysis to triangulate difference-in-differences

To triangulate difference-in-difference estimates with those of the mathematical model, we performed probabilistic bias analyses. Specifically, we assessed the influence of (1) nondifferential or differential outcome misclassification due to incomplete influenza testing among hospitalized patients and (2) an unmeasured time-dependent confounder of the relationship between the school-located influenza vaccination intervention and influenza hospitalization.

#### Bias analysis for outcome misclassification

Our original study estimated the impacts of school-located influenza vaccination on laboratory-confirmed influenza hospitalization. If some patients who truly had influenza were not tested for influenza, this could result in outcome misclassification with bias toward the null (Table 1). If testing proportions differed between the intervention and comparison sites, this could result in differential outcome misclassification and bias in an unknown direction. First, we corrected estimates of intervention impact on hospitalization for influenza on potential nondifferential misclassification due to incomplete influenza testing. We obtained estimates of the proportion of hospitalized patients with pneumonia and influenza International Statistical Classification of Disease codes who were tested for influenza from the CEIP, which provided influenza hospitalization data in the original study. These unpublished data were collected as part of CEIP’s evaluation of its activities in Alameda, San Francisco, and Contra Costa counties. It was not possible to stratify data by study site. First, we performed a probabilistic bias analysis. Based on CEIP data, we used a β(*α* = 44, *β* = 32) prior for the proportion of patients aged ≥ 65 years who were tested for influenza. This distribution has a mean of 0.58 and is concentrated between 0.38 and 0.78 (Table S1). The same prior distribution was used for intervention and comparison sites.

Next, to explore the influence of different testing proportions in the intervention and comparison sites, we corrected case counts for all combinations of testing probabilities from the prior distribution for each site in increments of 0.01. We corrected the number of observed hospitalized cases (*y_observed_*) in each study site for incomplete influenza testing using the formula *y_true_* = *y_observed_* × (1/*p*), where *y_true_* is the number of cases hospitalized with laboratory-confirmed influenza corrected for incomplete testing and *p* is the proportion of hospitalized patients tested for influenza. We assumed that the probability of testing positive was the same between patients who were and were not tested.

#### Bias analysis for unmeasured time-dependent confounding

The difference-in-difference analysis can remove time-invariant confounding, such as differences in vaccination coverage, insurance coverage, or underlying health status between the intervention and comparison sites that did not change over time. However, a key assumption of difference-in-differences is that there are no common shocks influencing both intervention and comparison groups that are independent of the intervention (ie, no time-dependent confounders). As one example of such a time-dependent confounder, we hypothesized that the implementation of the main provisions of the Affordable Care Act (ACA), which coincided with the start of the intervention in 2014, could have changed health insurance coverage over time differentially between sites. Site- and time-specific changes in health insurance could also have resulted in vaccination coverage or health status of adults 65 years or older that were differential between sites and unrelated to the intervention. If the ACA increased insurance coverage (and, in turn, vaccination and health status) in the intervention site but not the comparison site during the intervention, this would have resulted in bias away from the null (Table 1). Conversely, if ACA had increased coverage in the comparison site but not the intervention site, this would have resulted in bias toward the null.

We used bias analysis to correct estimates for a single unmeasured, time-dependent confounder. We define *Y* as the outcome, *X* as an indicator for the intervention vs comparison site, and *C* as an indicator for the unmeasured confounder (health insurance coverage). Priors for correcting for an unmeasured confounder include (1) the probability of the confounder in the intervention group P(*C*|*X* = 1), (2) the probability of the confounder in the comparison group P(*C*|*X* = 0), and (3) difference in risk between hospitalization (*Y*) and the confounder (*C*) in the comparison group (*X* = 0), assuming no effect modification by *X* ($E\left[Y|C=1,X=0\right]-E\left[Y|C=0,X=0\right]$).^19^ We defined priors separately for the period before and during the intervention to allow for time-dependent confounding. We defined both realistic and alternative, less realistic priors to investigate what priors would be required to replicate the mathematical model’s findings (see details in Appendix S1).

We calculated 3 Mantel-Haenszel risk differences (RD_MH_) correcting for confounding: (1) the risk difference for the intervention in the preintervention period (RD_MH, pre_), (2) the risk difference for the intervention during the intervention (RD_MH, post_), and (3) the risk difference comparing the risk during vs before the intervention in the comparison group (RD_MH, comparison_).^19^ We calculated the difference-in-difference correcting for bias as RD_MH, post_ – RD_MH, pre_ and the relative reduction in the difference-in-difference correcting for bias as (RD_MH, post_ – RD_MH, pre_)/RD_MH, comparison_.

In all bias analyses, we randomly sampled bias parameters from prior distributions and repeated analyses 10 000 times to obtain distributions of bias-corrected estimates. We used a bootstrap with 1000 replicates to obtain credible intervals for the ratio of mean prior values for each prior. We calculated the bias simulation interval as the 2.5th and 97.5th percentiles of the distributed of bias-corrected estimates.^19^

### Mathematical modeling

**Figure 2:**
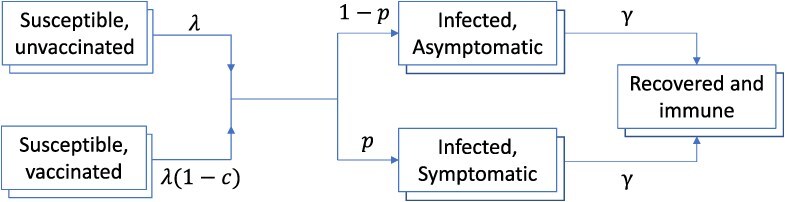
Schematic illustration of the mathematical model. We distinguish those who were vaccinated in time for the 2017-2018 season (lower panel) from those who were not (upper panel). Model symbols are as follows: force-of-infection $\left(\lambda \right),$ vaccine efficacy in reducing the force-of-infection $(c)$, proportion of infections that are symptomatic $(p)$, and per-capita recovery rate $\left(\gamma \right)$. “Layers” to each compartment denote stratification into 6 age groups: < 4, 5 to 11, 12 to 17, 18 to 49, 50 to 64, and $\ge$65 years old.

We modeled the indirect effect of the intervention among adults $\ge$ 65 years of age using an age-structured, deterministic, compartmental model, illustrated schematically in Figure 2. We focused on influenza A, which was responsible for 72% of virologically confirmed influenza infections during the 2017-2018 season in California. For simplicity, we did not distinguish H3 and H1 subtypes of influenza A.^21^ The model was stratified into 6 different age groups: < 4, 5 to 11, 12 to 17, 18 to 49, 50 to 64, and $\ge$ 65 years. To capture mixing between different age groups, we drew from contact matrices recently estimated for the United States.^22^ The overall structure shown in Figure 2 was further stratified by influenza vaccination status, distinguishing those who did and did not receive seasonal influenza vaccination during the 2017-2018 season. Given the focus of this analysis on indirect effects, we assumed that the measured vaccine efficacy is against infection and, moreover, that vaccination confers protection through a “leaky” mechanism: that is, we assumed that all receiving influenza vaccination have their risk of infection reduced by an amount equivalent to the vaccine efficacy. Because the majority of seasonal vaccination coverage in the United States is typically completed before the onset of the influenza season,^23^ we modeled age-specific vaccination simply through initial conditions, ensuring that the initial population vaccinated compartments reflected the estimated, age-specific vaccination coverage.

#### Data and calibration

We calibrated model parameters to match the available epidemiologic data in the control district (West Contra Costa). In particular, as a proxy for incidence, we used district-level data from the CEIP for age-specific, weekly hospitalizations that were virologically confirmed as being influenza. These are the same data that were used to estimate indirect effects in the cohort study. To link these data with the weekly incidence of symptomatic influenza in the community, we drew from previous estimates by the CDC for the age-specific proportions of symptomatic influenza cases that are hospitalized, tested for influenza, and reported to FluSURV-NET.^24^ These multipliers are available by age group, but only at the national level. Because data on influenza vaccination coverage by site were not available, we drew from state-level estimates of vaccination coverage in California, assuming coverage would have been the same in the absence of the intervention (see Figure S1A) (C. Reed, personal communication, August 31, 2020). We drew from national-level estimates of vaccine efficacy against influenza A for the 2017-2018 season (see Figure S1B). Further details on model calibration are provided in Appendix S2.

#### Intervention modeling

We simulated the impact (reduction in influenza hospitalizations among those aged $\ge$ 65 years) of the previously estimated increase in influenza vaccination coverage among the 5- to 11-year age group in 2017-2018.^10^ Specifically, we simulated an increase in vaccine coverage from 53% to 64%, the increase in vaccination observed in 2017-2018 in the Shoo the Flu evaluation.^10^ Although in theory, the intervention may have also influenced other age groups to increase uptake of vaccination, we had no data to this effect: thus, we assumed no change in vaccination coverage in other age groups.

### Sensitivity analyses to triangulate the mathematical model

We performed sensitivity analyses to identify mathematical model parameters that would best explain differences in the results of the transmission model and the cohort study. We expected that several possible sources of bias in the mathematical model could have resulted in bias toward the null (Table 1). This includes underestimating the contact rate between schoolchildren and the elderly in the intervention site, overestimating preexisting immunity to the circulating influenza strain, overestimating duration of infection and underestimating infectiousness, and underestimating the proportion of symptomatic individuals and infectiousness of asymptomatic cases. To assess these potential biases, we performed an alternative, “constrained” calibration, one where—in addition to the data described above—we also included the requirement that model projections should capture the observed reduction in hospitalization in those $\ge$ 65 years of age from the difference-in-difference analyses.

Replication scripts are available at https://github.com/jadebc/SLIV-modeling-bias.

## Results

### Difference-in-difference analysis

In the original study, the difference-in-difference comparing influenza hospitalizations in the intervention and comparison areas among adults $\ge$65 years was –160 per 100 000 (95% CI, –267 to –53) in 2017-2018 (Table 2).^10^ This was equivalent to a relative reduction in influenza hospitalization incidence of 22% (8%-37%).

**Table 2:** Results from triangulation analyses.

**Analysis**	**Relative reduction (CI** ^**a**^ **)**	**Difference-in-difference per 100 000 (CI** ^**a**^ **)**
*Difference-in-difference analysis*		
Original analysis	22.3% (7.6% to 37.1%)	–160 (–267 to –53)
Bias analysis for outcome misclassification	28.6% (27.5% to 29.9%)	–277 (–341 to –225)
Bias analysis for time-dependent confounding		
Realistic scenario	28.7% (26.9% to 30.6%)	–160 (–169 to –150)
Extreme values of health insurance coverage by site	26.5% (–59.0% to 104.1%)	–157 (–613 to 294)
Extreme values of the relationship between hospitalization and health insurance coverage	27.8% (24.0% to 31.4%)	–159 (–177 to –140)
*Mathematical model*		
Main analysis	2.1% (0.40% to 5.0%)	—^b^
Sensitivity analysis with constrained model	20.0% (18.0% to 21.4%)	—^b^

Relative reductions were calculated as the percent reduction in cumulative hospitalizations in those over 65 years old arising from increasing vaccination in schoolchildren. Difference-in-difference models quantified the difference in incidence in the intervention district before the intervention and during the intervention minus the difference in incidence in the comparison district before and during the intervention. For the bias analysis, estimates are the mean across all replications, and the bias simulation interval is the 2.5th and 97.5th quantile across replications. All bias analyses included 10 000 replications except for analyses of extreme values of the relationship between hospitalization and health insurance coverage, which had 3615 simulations, excluding those that resulted in zero case counts. In the constrained model, we required projections to replicate the indirect effect estimate from the cohort study by incorporating in the posterior density a likelihood term for the impact of vaccination in those $\ge$65 years of age.

a CI indicates 95% confidence interval for the original difference-in-difference analysis, the bias simulation interval for the bias analysis, and the Bayesian credible interval for the mathematical models.

b Difference-in-difference estimates are not presented for the mathematical model because they were fit to data from the 2017-2018 season only.

### Mathematical model

**Figure 3:**
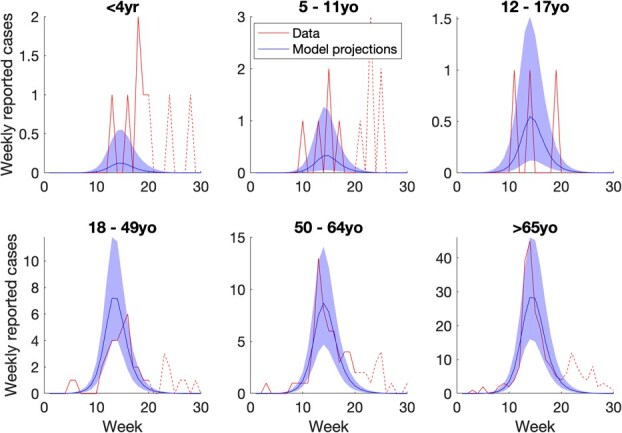
Results of model calibration to epidemiologic data in the control district (West Contra Costa). Curves in red show age-specific data on virologically confirmed influenza hospitalizations from FluSURV-NET. Solid red lines show parts of the epidemic when influenza A dominated (to which the data were calibrated) while dashed red lines show parts of the epidemic driven by influenza B (not addressed in the calibration). Blue curves show best model fits, scaled by multipliers associating the data with the incidence of symptomatic influenza, with shaded regions showing 95% Bayesian uncertainty intervals. While this figure shows model fits to the weekly data, Figure S2 in the supporting information also shows model fits to cumulative reported cases. The x-axis notes weeks during the influenza season for 2017-2018, which spanned the period from October 21, 17, to April 28, 2018.

The mathematical model captured a reasonable fit with the dynamics of influenza hospitalization in the 3 oldest age groups in the control district; data were sparse for younger age groups (Figure 3). Figure S2 shows model fits to cumulative numbers hospitalized, again showing a reasonable fit, while Figure S3 illustrates the marginal posterior distributions for each of the model parameters. For an intervention that increased influenza vaccination coverage in schoolchildren 5 to 11 years old from 58% to 69%, the model estimated a 2.1% reduction (95% Bayesian credible interval [CrI], 0.40%-5.0%) in hospitalizations in those $\ge$65 years of age, substantially lower than the empirically observed reduction of 22%.

### Triangulation using probabilistic bias analysis for the difference-in-difference model

#### Outcome misclassification

Using probabilistic bias analysis to correct for nondifferential misclassification due to incomplete influenza testing of patients hospitalized for pneumonia and influenza, the mean of difference-in-differences across replications was –277 per 100 000 (bias simulation interval [BSI], –341 to –225). This was equivalent to a relative reduction of 28.6% (BSI, 27.5%-29.9%). The larger effect estimate suggests that the original difference-in-difference estimate may have been underestimated due to outcome misclassification.

**Figure 4:**
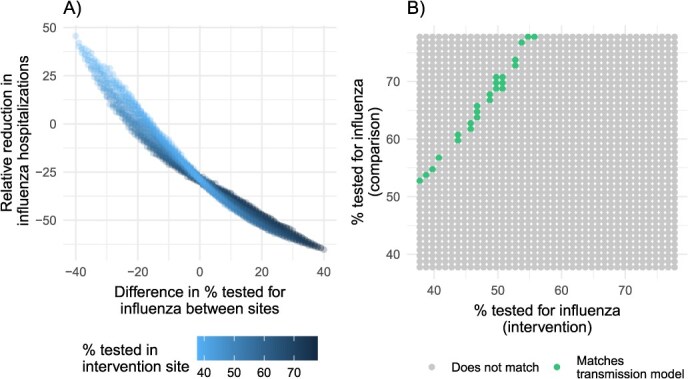
Concordance of epidemiologic and mathematical models by the percentages of patients tested for influenza in the intervention vs the comparison site. Using probabilistic bias analysis, we corrected empirical estimates of the incidence of laboratory-confirmed influenza hospitalizations in the study site for misclassification due to potential incomplete influenza testing of patients hospitalized for pneumonia and influenza. In an exploratory analysis, we investigated all possible combinations of priors in each site. (A) Shows relative reductions in influenza hospitalization incidence by the percentage of patients tested for influenza in the intervention site and the difference in the percentage tested between sites. (B) Shows whether bias-corrected estimates matched empirical estimates by the percentage of patients tested for influenza in the intervention and comparison sites.

Next, we attempted to replicate impact estimates from the mathematical model by repeating analyses that corrected for outcome misclassification using all possible combinations of priors and including the possibility of differential misclassification. Only 1.4% of estimates that corrected for outcome misclassification aligned with the mathematical model estimates. Bias-corrected relative reductions were similar to those from the mathematical model when influenza testing was approximately 15% to 23% lower in the intervention site than in the comparison site (Figure 4A) and when the percentage tested for influenza in the intervention site ranged from 38% to 56% (Figure 4B).

#### Unmeasured confounding

Using probabilistic bias analysis to correct for potential unmeasured time-dependent confounding, results were similar to the original analysis; the mean of difference-in-differences across replications was –160 per 100 000 (BSI, –169 to –150), and the mean percentage change was –28.7% (BSI, 26.9% to 30.6%) (Table 2). No estimates aligned with the results of the mathematical model. In the alternative analysis with more extreme prior values of health insurance coverage by site, the mean of difference-in-differences across replications was –157 per 100 000 (BSI, –613 to 294), and the mean percentage change was –26.5% (BSI, –59.0% to 104.1%) (Table 2). Only 1% of bias-corrected estimates aligned with the mathematical model (Figures S3-S4); this occurred under extreme shifts in insurance coverage during the intervention period (from approximately 0%-90% in the intervention site and from approximately 90%-0% in the comparison site) and when the effect of health insurance coverage on influenza hospitalizations was weaker before vs during the intervention period. In the alternative analysis with stronger relationships between hospitalization and health insurance coverage, no results aligned with the mathematical model; the mean of difference-in-differences across replications was –159 per 100 000 (BSI, –177 to –140), and the mean percentage change was –27.8% (BSI, 24.0% to 31.4%) (Table 2).

### Triangulation using sensitivity analyses for the mathematical model

To examine which parameters were most strongly associated with the low impact projected by the mathematical model, we performed the “constrained” calibration described above. Table S2 shows estimated model parameters, and Figures S5 to S7 show the results of this calibration, in terms of parameter estimates and agreement with the epidemic dynamics. Table 2 illustrates that this model indeed approximates the expected indirect effect in those $\ge$ 65 years of age.

**Figure 5:**
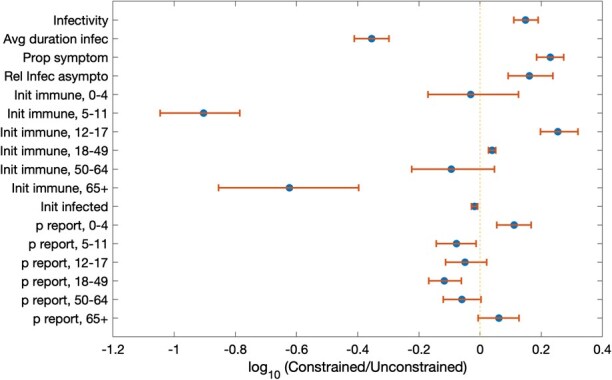
Comparison of parameter estimates between “constrained” and “unconstrained” calibrations. In constrained calibration, we incorporated in the posterior density a likelihood term for the impact of vaccination in those $\ge$ 65 years of age. The figure shows a comparison between parameter values, constrained vs unconstrained. The comparison was calculated using bootstrapped means as follows: for each parameter value, we drew a sample of 25 parameter values at random from the posterior density and calculated the sample mean. The values plotted here show the ratios of sample means thus obtained (a ratio of constrained vs unconstrained models) and their uncertainty, when iterated 1000 times. The vertical dashed line corresponds to a ratio of 1 (hence a log-ratio of zero); uncertainty intervals show 95% Bayesian credible intervals. Abbreviated y-axis labels are as follows: “Avg duration infec” denotes the average duration of infectivity; “prop symptom” denotes the proportion of infections that are symptomatic; “Rel Infec asympto” denotes the relative infectiousness of asymptomatic cases, relative to those who are symptomatic; “Init immune” denotes the proportion of each age group that were initially immune, before the vaccination programme; and “p report” denotes the proportion of all symptomatic cases that were reported to FluSURV-NET.

Figure 5 shows results of a comparison of marginal posterior samples in the unconstrained and constrained calibrations, plotting the ratio of bootstrapped means between the two. Parameters whose uncertainty intervals cross 0 on the logarithmic axis are those for which there appears to be no systematic difference between constrained and unconstrained posterior densities. Among the parameters that do show a systematic difference, 2 notable examples are the proportion initially immune in those aged 5 to 11 years and in those $\ge$ 65 years of age. In particular, in order to capture the impact of vaccination, the constrained calibration systematically estimates these parameters as having lower values than in the unconstrained calibration: that is, the mathematical model requires preexisting immunity in those aged 5 to 11 years to be 5.6% (95% CrI, 0%-15%) and in those $\ge$65 years of age to be 2.3% (95% CrI, 0%-11%), in order to capture indirect effects in $\ge$65-year-olds. For comparison, these levels of immunity in the unconstrained model are, respectively, 47% (95% CrI, 14%-80%) and 12% (95% CrI, 0%-37%). Among other parameters showing a systematic difference between the samples are the rate of infection (systematically estimated as higher in the constrained model), the average duration of infectiousness (lower in the constrained model), and the proportion symptomatic (higher in the constrained model).

## Discussion

We estimated the indirect effect of a city-wide school-located influenza vaccination program on influenza incidence among individuals 65 years and older using triangulation of 2 different analytic strategies. While the original difference-in-difference analysis^10^ found that school-located influenza vaccination was associated with a 22.3% relative reduction in community-wide influenza hospitalizations among adults $\ge$ 65 years old, a mathematical model that posed the same question using the same underlying data found a relative reduction of only 2.1%. When we investigated plausible sources of bias in the difference-in-difference analysis using probabilistic bias analysis with realistic assumptions, relative reductions were similar or even larger than in the original study. We were only able to align results of the mathematical model and difference-in-difference models in a small number of unrealistic bias scenarios. Sensitivity analyses of mathematical models showed that model parameters replicating the original study results would require unrealistically low levels of preexisting immunity to influenza among school-age children and older adults. Other factors included a higher rate of influenza infection, a lower average duration of influenza infection, and a higher proportion of symptomatic individuals. Mathematical modeling offers a tool for capturing complex transmission dynamics in a systematic way: in doing so, however, such models introduce many more assumptions than in a difference-in-difference analysis. The influence of these assumptions can be difficult to thoroughly evaluate, even in detailed sensitivity analyses. Considering the relative strengths and weaknesses of each approach, we conclude that the intervention likely produced indirect effects among older adults but that the true effects may have been somewhat smaller than the estimates published in the original study.

The modeling analysis also yielded a markedly more modest impact than previous modeling studies of vaccinating schoolchildren against influenza. These differences are likely due to the comparators involved: a 2009 study in the United States^13^ and a 2013 UK study^3^ both modeled an increase in vaccination coverage among children from 0% to 70%. Vaccination coverage in both the United States and the United Kingdom has increased substantially in recent years: in the present study, we modeled an increase in influenza vaccination coverage in schoolchildren from 58% to 69%. High levels of preexisting immunity in schoolchildren would tend to reduce the incremental impact of additional vaccination coverage in this group. Likewise, high levels of preexisting immunity in the elderly would reduce the extent to which they can benefit from indirect effects of vaccination in other age groups. Contrary to the low levels of immunity required by the constrained model, studies in the United States and elsewhere have shown the presence of antibodies to influenza in over 40% of school-aged children.^25-27^ Although some of these studies were performed in the context of the 2009 influenza pandemic, we might expect the prevalence of antibodies to seasonal influenza to be comparable or greater, arising from several seasons of exposure to seasonal influenza viruses as well as vaccination in past seasons.

Difference-in-difference analyses aligned with the mathematical model only when the percentage of patients $\ge$ 65 years old who were tested for influenza was 15% to 23% lower at hospitals in the intervention area vs comparison area. While it was not possible to verify testing proportions by site, we consider this difference in testing proportions to be plausible because it is within the range of observed testing proportions, which varied from 21% to 92%, depending on the provider type and age group during the study period. The California Department of Public Health mandates reporting of laboratory-confirmed influenza deaths among individuals 0 to 64 years of age but not influenza hospitalizations. Whether a physician orders an influenza test for a patient likely depends on a range of factors that could plausibly vary between study areas, including hospital protocols, laboratory capacity, patient insurance type, patient risk factors, patient symptoms, and the severity of circulating influenza strains at a given time.

The analysis for possible unmeasured time-dependent confounding due to the start of the ACA did not explain the discrepancy in the mathematical model and the epidemiologic study’s findings. Confounding-corrected estimates aligned with mathematical models only when we assumed extreme, unrealistic changes in insurance coverage that were in the opposite direction between study sites. Most studies have not found evidence that the ACA led to increased influenza vaccination,^28-31^ and CDC estimates do not show increased national influenza vaccination coverage from 2014 to 2018 in any age group.^32^ Here, we focused on adults $\ge$ 65 years old who were already covered by Medicare, and lower preventive service and premium costs under ACA may not have been substantial enough to influence the risk of influenza hospitalization.

We identified 3 potential hypotheses that could be tested to further triangulate the results of the difference-in-difference model and mathematical model, but these require additional data (Table 3). The first hypothesis is that the mathematical model underestimated the impact of the intervention because there were substantially lower levels of protective immunity to the predominant circulating strains among schoolchildren and the elderly than estimated by the model. Seroprevalence surveys in these age groups could have been used to assess baseline immunity. The second hypothesis is that in the intervention site, exposure of the elderly to schoolchildren was substantially higher than modeled. Detailed contact surveys in the population studied could be used to assess contact patterns. Finally, the third hypothesis is that the difference-in-difference analysis overestimated the intervention effect because the proportion of people tested for influenza was substantially lower in the intervention than the comparison site. Systematic data on testing rates by study site could be used to assess this.

**Table 3:** Summary of testable hypotheses to reconcile difference-in-difference analysis and mathematical modeling.

**Hypothesis**	**Data required to test hypothesis**
*Mathematical modeling underestimates intervention impact*	
Substantially lower levels of protective immunity to circulating strain, among schoolchildren and the elderly, than estimated by the model	Seroprevalence surveys in relevant age groups
In population receiving the intervention, exposure of the elderly to schoolchildren is substantially higher than modeled	Contact surveys specific to the populations in the study
*Difference-in-difference analysis overestimates intervention impact*	
Proportion of people tested for influenza was substantially lower in the intervention than control site	Systematic data on testing rate by study sites

See discussion for further details. While the current study did not have the data necessary to test these hypotheses, this list serves as a summary of data that would be helpful to collect in any future such studies, to better address any similar discordance between model-based and difference-in-difference analysis.

This study was subject to several limitations. The mathematical model could not explicitly model household contact structure, relying instead on contact matrices to capture intergenerational mixing. Future work could compare the results using individual-based models, which can capture household contact structure more explicitly, as well as potentially incorporating heterogeneous mixing by race/ethnicity and other groupings (data that were not available in the present study). Additionally, the uncertainty analysis of our mathematical model focused on model parameters such as prior immunity but did not examine the effect of alternative model structures (“structural” uncertainty), including alternative ways of modeling vaccine-induced immunity. For age-specific vaccination coverage in the comparator scenario (ie, in the absence of intervention), we assumed this would be the same as coverage at the state level. If, in reality, vaccination coverage under this scenario would have been substantially lower, then the estimated intervention impact would be correspondingly greater than modeled here. However, analogously to the results shown in Figure 4, we expect that such reductions in baseline coverage would have to be implausibly large in order to align with the impact estimated in the difference-in-difference analysis. Another assumption, again for lack of site-specific data, was that reporting rates were the same between intervention and control districts. If indeed the true reporting rates were substantially higher in the intervention district, this would suggest that the underlying epidemic was correspondingly smaller, thus having the potential for greater impact of a given coverage of vaccine (and vice versa). Finally, in the model, the sensitivity analysis in Figure 4 focused on marginal parameter distributions and thus does not address the potential role of correlations between estimated parameters. This was conducted for simplicity: nonetheless, one area for future work would be to perform a more systematic analysis of the multivariate correlations underlying the difference between the 2 models.

Our probabilistic bias analysis was sensitive to the assumed priors. We have higher confidence in our prior definitions for influenza testing proportions since they were based on local testing proportion estimates; priors for time-dependent confounding analyses were based on published studies from other populations that may not resemble values in our study population. However, our estimates of influenza testing proportions included some surveillance areas outside of the study site, so priors may be inaccurate, and we did not have data on the relative difference in testing proportions between study areas. Data were not available to assess whether patients who were tested had a higher probability of testing positive than those who were not. Finally, we conducted this study in a single influenza season with relatively high transmission (2017-2018); direct or indirect effects of school-located influenza vaccination would be expected to be lower in absolute magnitude in seasons with lower influenza transmission.^10^

Our approach highlights the value of using mathematical models together with empirical epidemiologic studies of infectious diseases to explore potential sources of bias through triangulation. In addition, in future studies, conducting seroprevalence surveys, contact surveys, and assessments of diagnostic testing practices would support triangulation analyses for infectious diseases.

## Supplementary Material

Web_Material_kwae365

## Data Availability

Influenza hospitalization data may be requested from the California Emerging Infections Program (info@ceip.us; https://ceip.us/contact/).
